# Flux of transcript patterns during soybean seed development

**DOI:** 10.1186/1471-2164-11-136

**Published:** 2010-02-24

**Authors:** Sarah I Jones, Delkin O Gonzalez, Lila O Vodkin

**Affiliations:** 1 Department of Crop Sciences, University of Illinois, Urbana, IL 61801, USA; 2 Current address: Dow AgroSciences, Indianaoplis, IN 46268, USA

## Abstract

**Background:**

To understand gene expression networks leading to functional properties of the soybean seed, we have undertaken a detailed examination of soybean seed development during the stages of major accumulation of oils, proteins, and starches, as well as the desiccating and mature stages, using microarrays consisting of up to 27,000 soybean cDNAs. A subset of these genes on a highly-repetitive 70-mer oligonucleotide microarray was also used to support the results.

**Results:**

It was discovered that genes related to cell growth and maintenance processes, as well as energy processes like photosynthesis, decreased in expression levels as the cotyledons approached the mature, dry stage. Genes involved with some storage proteins had their highest expression levels at the stage of highest fresh weight. However, genes encoding many transcription factors and DNA binding proteins showed higher expression levels in the desiccating and dry seeds than in most of the green stages.

**Conclusions:**

Data on 27,000 cDNAs have been obtained over five stages of soybean development, including the stages of major accumulation of agronomically-important products, using two different types of microarrays. Of particular interest are the genes found to peak in expression at the desiccating and dry seed stages, such as those annotated as transcription factors, which may indicate the preparation of pathways that will be needed later in the early stages of imbibition and germination.

## Background

**Figure 1:**
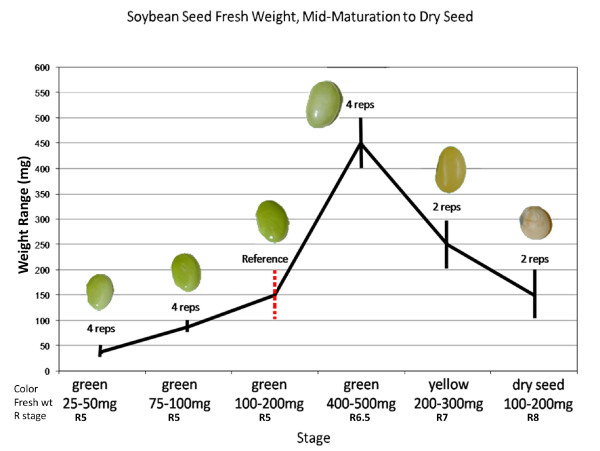
**Timeline of development in soybean seeds from mid-maturation to desiccation**. Fresh weight range in mg shown on Y axis. Dotted bar indicates the reference tissue. Reproductive (R) developmental stages according to [1] shown below each stage are approximate.

During the mid-maturation stage of soybean (*Glycine max*) seed development, the majority of the nutrients required for early seedling growth are acquired; many of these substances, such as oils and proteins, are of agronomic importance as well. Soybean seeds first begin to form on the plant at the stage known as R3, when the parent plant has 11-17 leaf nodes [1]. Between the stages of R3 and R7 the seeds grow rapidly, accumulating nutrients like carbon and nitrogen and storage proteins such as glycinin and alpha- and beta-conglycinin [1-3]. See Figure 1 for a timeline of development of mid to late maturation seed stages in soybean. The stem and leaves of the parent plant begin to turn yellow during R6, with the older leaves senescing and dropping from the plant [1]. By R7, the seed's accumulation of dry weight is almost complete, and the uptake of nutrients slows down [1]. The peak fresh weight of the cultivar Williams, used here, is about 400-500 mg; the seed contains about 60% moisture at this point [1]. As the seeds begin to dry and turn yellow, they become capable of germinating; however, most are as yet unable to support seedling growth [1,4]. Developmental processes in the seed come to an end and the embryo prepares for desiccation [4]. As water is lost, the total fresh weight of the seed decreases; the seed coat vascular system is crushed and disappears [5]. By R8, most of the plant, including the pods and seeds, is brown and dry [1]. Most seeds are able to both germinate and sustain seedling growth at about 55% moisture; however, several more days of drying may be required before they reach the best moisture content for harvest, around 15% [1,4]. Due to the enzymes, ribosomes, initiation and elongation factors, and other compounds that were produced during development and stored in the seed, metabolic activity can resume almost immediately upon imbibition of water [2].

In 2000, Girke et al. [6] identified a number of seed-specific genes in *Arabidopsis *using microarrays created with 2600 cDNAs derived from seeds. About 260 genes, or 10% of those studied, were found to have at least ten-fold higher expression in the seeds than in the roots or leaves. Most of these seed-specific genes encoded the expected seed storage proteins as well as transcription factors and genes of unknown function. Overall, this study provided the first available expression data on thousands of *Arabidopsis *genes from both seeds and other tissues. Ruuska et al. (2002) [7] expanded on this work by studying the expression levels of >3500 seed-specific *Arabidopsis *genes over five time points. These time points included the stages of major storage reserve accumulation and ended just before seed desiccation. Approximately 1525 of these clones were found to have a significant expression level change during seed development. Results indicated that genes in the same metabolic pathway could show different expression patterns, suggesting they were regulated by different factors. This differential regulation might be coordinated with shifts from starch to oil and protein accumulation, and the contrasting expression patterns of very similar genes could indicate the movement of carbon from one part of the cell to another during the synthesis of metabolites like fatty acids.

More recently, Liu et al. (2008) [8] performed a comprehensive study of maize kernel development from early embryogenesis through storage product accumulation and desiccation, using arrays containing more than 30,000 unique maize genes. More than 10% of the genes were found to be significantly differentially expressed (p < 0.01) in at least one stage studied, with the highest number of differentially expressed genes occurring during the phase of beginning deposition of storage materials. Most of the 3400 significant genes were up-regulated (compared to the consecutive phase) during the middle three phases, but most of these genes were down-regulated during the first phase (cell division) and the last phase (desiccation). Additionally, genes such as LEA proteins, seed maturation proteins, and those related to ethylene signaling, including some ethylene-related transcription factors, were found to increase in expression at the last two mature and desiccating stages, compared to their expression at the youngest stage.

In this study, data obtained about gene expression changes in soybean seeds during mid-maturation to desiccation were evaluated at five different time points using soybean cotyledons. The expression changes in selected genes of interest were also tested using a second microarray format in which genes were spotted many times on the same slide, to provide additional replicates and thus statistical power compared to other methods such as RNA blotting for verification of gene expression. Studying the broad patterns of gene expression change in these later stages of seed development can yield important insights into the processes of seed filling, desiccation, and preparation for quiescence and germination.

## Results & Discussion

### Data collection and p-value analysis

Two different array formats were used for different aims. Array Format 1 provided a global view of gene expression trends during seed development as it consists of a low redundancy set of 27,609 soybean cDNAs from a variety of soybean tissues. See [9] for more details of the unigene selection and of the microarray construction. Both the 5' and the 3' ends of the cDNAs were annotated using the top BLAST hit (e-value ≤ 10^-6^). Format 2 consists of 192 oligos designed from the cDNAs of Array Format 1 and spotted forty times each on a single slide in order to validate expression for the selected cDNAs with a high number of within-slide replicates. Both the cDNAs of Array Format 1 and the oligos of Array Format 2 will likely detect mRNAs of all family members with 85% similarity and thus are not likely to distinguish all family members or paralogous genes [9].

For Array Format 1, total RNA was extracted from soybean cotyledons taken from seeds in the following fresh weight ranges: 25-50 mg, 75-100 mg, 400-500 mg, and 200-300 mg with yellow-colored tissue, as shown in Figure 1. Total RNA was also extracted from whole dry soybean seeds at a weight of 100-200 mg. Each of these five stages was compared to total RNA extracted from soybean cotyledons taken from seeds in the 100-200 mg fresh weight range, which was considered the reference tissue. For Array Format 2, only three of the stages were used (25-50 mg, 400-500 mg, dry seed) but were compared to the same reference tissue (100-200 mg cotyledon).

The program GeneSpring (Silicon Genetics, Redwood City, CA) was used to analyze the data from all experiments. Using Array Format 1 with five stages of soybean cotyledon development compared to the same reference tissue, 2227 genes were found to have p-values ≤ 0.05 in at least three of the five stages of development. Thus their expression levels in the experimental tissue were significantly different from their expression levels in the reference tissue in at least three of the five stages.

**Figure 2:**
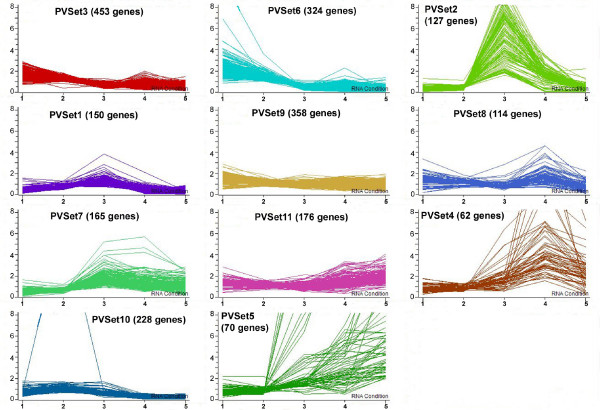
**The expression profiles of 2227 soybean cDNAs in cotyledons, divided into eleven k-means clusters (PVSets)**. All of these genes have p-values ≤ 0.05 in at least three of the five stages. Normalized ratio intensities are shown on the Y axis. The five stages of development are marked on the X axis, from youngest to oldest (left to right): 1 = 25-50 mg, 2 = 75-100 mg, 3 = 400-500 mg, 4 = yellow, 5 = dry seed. Sets are numbered randomly by GeneSpring. Set numbers and the total number of genes per set are shown at the top of each set.

The 2227 genes were divided into eleven *k*-means clusters (here called PVSets, to indicate the p-value restriction) based on the similarity of their expression profiles (Figure 2). The *k*-means algorithm, used here by GeneSpring, randomly separated the genes into the number of clusters defined by the user (in this case, eleven). The centroid of each cluster was calculated by averaging the coordinates attached to each gene. Each gene was then reassigned to the centroid to which it was closest and the coordinates of the centroids were recalculated. This operation was performed numerous times until the data converged, resulting in the clusters shown. The cDNAs in the clusters were also divided into ten functional categories (Table 1) according to their annotations, which were based on the top BLAST hit (e-value ≤ 10^-6^) and on the *Arabidopsis *gene ontology (TAIR). Table 1 also summarizes the percentages of genes in each functional category for five of the clusters.

**Table 1:** Percentage of genes in each of the ten functional categories in five PVSets

Category Name	Gene Examples	PVSet1	PVSet2	PVSet4	PVSet6	PVSet11
Cell Growth & Maintenance	Tubulin, auxin-regulated, histone	26.3%	40.0%	39.3%	29.8%	25.4%

Energy	Chlorophyll binding, RuBisCO	3.0%	1.7%	1.8%	27.7%	6.5%

Hypothetical/Unknown Function	Hypothetical/unknown function in databases	24.1%	35.0%	25.0%	17.4%	32.5%

Other (Miscellaneous)	Transposons, cell death, pollen-related	1.5%	1.7%	1.8%	1.1%	0.6%

Oxidative	Metallothionein, cysteine protease, peroxidase	0.8%	2.5%	3.6%	0.7%	0.6%

Stress, Defense, Shock-related	Chitinase, drought resistance, stress-induced	4.5%	5.8%	8.9%	2.5%	8.9%

Signaling	Cytochrome P450, protein kinases, calmodulin	9.0%	5.8%	8.9%	10.3%	5.3%

Seed Proteins	Lipoxygenase, seed maturation protein, trypsin inhibitor	16.5%	0.0%	0.0%	5.0%	0.0%

Transcription	DNA-binding, transcription factors, zinc finger proteins	13.5%	5.8%	3.6%	3.9%	13.0%

Transporters and Membrane Proteins	Sugar/amino acid transporters, membrane intrinsic proteins	0.8%	1.7%	7.1%	1.8%	7.1%

The ten functional categories are shown along with examples of gene families placed in each category. The percentage of genes in each functional category is shown in each of five PVSets.

Here we focus on a selection of the data of particular biological interest, involving genes which peak in expression at specific stages of development and on genes in specific functional categories such as cell growth and maintenance, energy, and storage proteins. We also concentrate on some transcriptional factors whose expression increases during the latter stages of seed maturation.

### Genes related to cell growth and maintenance, and signaling

Many of the cDNAs found in a single cluster have similar annotations, and the expression patterns of many related cDNAs are consistent with known biological processes in seed development. For example, PVSet6 (peak at the 25-50 mg stage) contains an unusually large number of cDNAs with annotations related to tubulins (both alpha and beta), histones, and chaperones (about 37% of the total *Cell Growth and Maintenance *genes). Additionally, this same cluster contains cDNAs with annotations involved in fatty acid synthesis such as enoyl-ACP reductase and 3-ketoacyl-ACP reductase; and those related to cell walls and the cytoskeleton, such as cinnamyl-alcohol dehydrogenase and beta scruin. This set also has a high percentage of genes in the *Signaling *category, with annotations including products such as annexin, cytochrome P450, nucleoside diphosphate kinase, and protein phosphatase. The cDNAs in this set are most highly expressed at the youngest stage of development studied (25-50 mg) and likely indicate the activity of processes necessary for the young seed to create and expand its cells as it grows. Similar results have been seen, for example, in the work by Gallardo et al. (2007) [10] on developing *Medicago *seeds, where it was found that both gene expression and protein abundance for cytoskeleton-related products such as actin and tubulin decreased from the stage of early seed fill until maturation and desiccation.

PVSet6, as mentioned, contains a large number of genes related to tubulin. Of the five sets in Table 1, only one other gene annotated as tubulin was found, in PVSet2 (peak at the 400-500 mg stage). PVSet2 also contains two genes annotated as expansin (involved in cell wall extension) as well as one gene each annotated as polygalacturonase and xyloglucan endo-transglycosylase, whose products break down cell wall components. No tubulin-annotated genes were found in PVSets 4 or 11, in which expression levels peak at the yellow and dry stages. Interestingly, a few other cell wall-related genes were found to be highly expressed at the final, dry seed stage, including a cellulose synthase, a cinnamyl-alcohol dehydrogenase (involved in lignin synthesis), and a pectinacetylesterase, which is involved in the breakdown of pectin in cell walls. These changes in which cell wall-related genes are highly expressed at different stages of development reflect the complex manner in which the cell wall must adapt to the development of the seed--growing, filling with storage products, then desiccating for dormancy--by synthesizing and degrading different components of the cytoskeleton.

### ADR genes change dramatically during development

The most common annotation among the *Cell Growth and Maintenance *genes in PVSet2 (peak at the 400-500 mg stage) is *ADR12*, an auxin down-regulated gene of unknown function. About 35% of the *Cell Growth and Maintenance *genes in this set are annotated as *ADR12*, with another 8% annotated as the related gene *ADR6*. Another family member, *ADR11*, is found repeatedly in PVSet6 (peak at the 25-50 mg stage). However, no *ADR *genes are found in either PVSet4 or PVSet11, which peak at the final two stages of development. These *ADR *genes were first described in 1980 by Baulcombe and Key [11] as having reduced RNA concentration following auxin treatment of soybean hypocotyls. Datta et al. (1993) [12] found that they display tissue-specific expression in soybean under endogenous auxin conditions, and that their decrease in expression due to increased auxin is also tissue-specific. This same study also found that these genes are differentially expressed in soybean tissues in response to light, with some genes being induced and others repressed by light in a tissue-specific manner. Additionally, Thibaud-Nissen et al. (2003) [13] found that *ADR12 *increases in expression in soybean somatic embryos as they develop on auxin-containing media, while multiple *ADR *genes were found to be over-expressed during various stages of post-germination soybean cotyledon development [14]. *ADR6 *predicts a protein of approximately 272 amino acids, while *ADR11*'s protein is predicted to contain about 151 amino acids and *ADR12*'s only about 41 amino acids [12]. Investigation of the expression patterns and function of the *ADR *gene family is ongoing to determine what role it might play in cell growth and development in seeds.

### Other genes expressed at stage of highest fresh weight

PVSet1 has an expression profile very similar to that of PVSet2, as both contain genes that peak in expression at the 400-500 mg stage. The genes in PVSet1, however, have a lower peak of expression at that stage of highest fresh weight. Interestingly, despite the similarity in the expression profiles, there are a number of differences in the types of genes found in the two sets. For example, PVSet1 contains no *ADR *genes, which are abundant in PVSet2, and does not have as many genes related to cell wall functions. PVSet1 also has several genes annotated as alcohol dehydrogenase, while PVSet2 has none, and there are more genes related to protein degradation (such as protease regulatory subunits and F-box proteins) in PVSet1. PVSet1 additionally has a much higher percentage of genes in the *Transcription *category (13.5%) than PVSet2 does (only 5.8%). Perhaps the most distinct difference between these two similarly-shaped sets, however, is found in the *Seed Proteins *category. PVSet2 has no genes in this category; but PVSet1 has 16.5% of its genes classified here. Almost all of these genes in PVSet1 are annotated as lipoxygenase, which is involved in the storage of nitrogen and the oxidation of polyunsaturated fatty acids in seeds [15,16]. According to Wilson (1987) [17], this enzyme increases in activity until ten days before maturation. Lipoxygenase may also accumulate in the seeds for later use in reactions during early shoot growth [18].

### Energy genes have higher expression in early development

The percentage of genes with annotations in the *Energy *category is fairly small in four of the five sets categorized, ranging from less than 2% in PVSets 2 and 4 to about 6.5% in PVSet11. However, in PVSet6 (peak at the 25-50 mg stage), *Energy *genes account for about 28% of the total genes. This category includes genes with annotations related to chlorophyll binding and the photosystems. Lee et al. (2002) [19] found that a number of genes encoding enzymes related to glycolysis in maize kernels and embryos have an expression profile that decreases steadily from a peak during the early stage of development. Similarly, genes associated with glycolytic enzymes such as sucrose synthase, triosephosphate isomerase, and enolase are found in PVSet6, which has a similar pattern. The energy-generating functions associated with these genes are most necessary when the seeds are young, green, and actively photosynthesizing and growing, and much less needed when the tissue has begun to yellow and desiccate.

### Genes over-expressed at final stages of development

In contrast to the expected patterns of expression, a considerable number of cDNAs (such as those in PVSets 4, 5, 8, and 11) are strongly expressed in the final two stages of development, when the tissue is yellow and desiccating and turning into the hard, dry seed. Many of these genes in PVSets 4 and 11, unsurprisingly, are related to protein degradation, such as ubiquitin-conjugating enzymes, proteases, and proteasome regulatory subunits. The products of these genes are useful for breaking down proteins no longer needed as the seed prepares for quiescence. However, genes related to a number of other cellular processes are found here, too, including those whose products are involved in amino acid metabolism (S-adenosylmethionine synthetase, diaminopimelate epimerase, betaine aldehyde dehydrogenase) and fatty acid synthesis (omega-3 fatty acid desaturase), and genes whose products are related to cell walls (cellulose synthase, pectinacetylesterase) and cell division (kinesin, *CDC48*). Additionally, genes with expression patterns that increase at the yellow and dry seed stages, compared to the reference, include those involved in flavonoid synthesis, such as chalcone synthase and 4-coumarate-CoA ligase. Three genes annotated as chalcone synthase, including *CHS7*, are found in PVSet4, with one *CHS *gene in PVSet5 and another in PVSet8--all sets with expression patterns that increase in either the yellow seed or dry, hard seed stage. The increase in expression of isoflavonoid synthesis-related genes, especially *CHS7 *and *CHS8*, at later stages of soybean embryo development was also seen by Dhaubhadel et al. (2007) [20]. Translation factors, chaperones, and other products associated with protein-protein interactions are also found among these genes' products, which could assist in creating properly-folded proteins during seed desiccation. The mRNAs for these factors or the proteins they encode may be produced late in seed development and then stored in the seed for use during the early stages of imbibition and germination.

PVSet11 also has a high percentage of genes (13%) in the *Transcription *category. These transcription factors (bHLH, ethylene response factor, auxin response factor), zinc finger proteins, ribonucleoproteins, etc., could also be related to the process of preparing transcripts in anticipation of germination. Interestingly, in their comparison of transcriptome and proteome data for developing *Medicago truncatula *seeds, Gallardo et al. (2007) [10] found a significant increase in the number of up-regulated transcripts, particularly those with annotations involved in transcription and RNA processing, at the mature, desiccating stage of seed development--but without a corresponding increase in the abundance of up-regulated proteins. They therefore speculate that the up-regulated transcripts "contribute to the stored mRNA pool used for protein synthesis during germination," a process also discussed in [21].

### Confirmation of expression of selected genes using an oligo array

**Figure 3:**
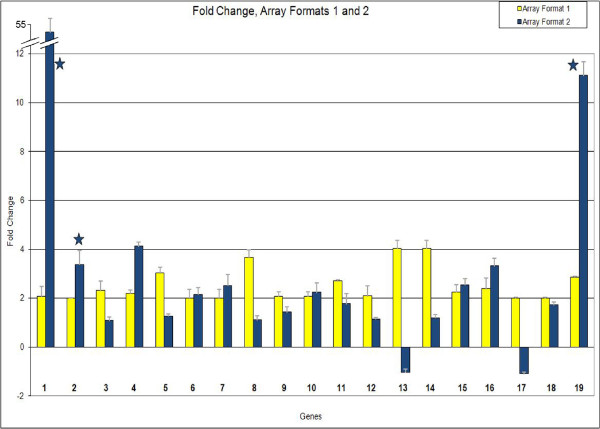
**Fold change for selected transcription factors in two array formats**. The stages dry seed vs. 100-200 mg cotyledon are compared. Y-axis shows fold change, X-axis shows nineteen transcription factors found to increase in expression at the dry seed stage using Array Format 1. Numbers along X-axis correlate to Table 2. Yellow bars represent fold change in Array Format 1; blue bars represent fold change in Array Format 2. Fold changes are based on normalized ratios. Stars indicate standard error of measurement is >0.5. Error bars show standard error of each measurement. The actual p-values and standard errors are shown in Table 2.

A different type of array, Array Format 2, was used to retest the expression levels of selected genes. This subset of 192 oligos includes genes annotated as storage proteins, cell wall proteins, transcription factors, and other genes of interest which were selected individually based on the results of the 27,000 cDNA microarray study and included transcription factors that appeared to be more prevalent during the latter stages of seed development. Oligos were designed for these 192 genes based on the cDNAs, and the oligos were spotted forty times each on the same array, for a total of 7680 spots. The forty spots representing one gene on the array were averaged together as replicates to provide greater statistical power, and two arrays were hybridized per stage of development, meaning that each ratio represents eighty measurements. In Figure 3, the fold changes of nineteen transcription factors from both array formats are displayed, at the final stage of dry seed (versus the reference). These nineteen transcription factors were chosen for further study on Array Format 2 based on their results in Array Format 1, i.e., they all had significantly increased expression at the dry seed stage compared to the reference. When the same stage is measured with Array Format 2, almost all of the transcription factors are shown to be over-expressed, consistent with the results from the previous array. Only three of the measurements on the graph have standard error above the threshold of 0.5 (starred), meaning most of the genes' measurements are repeatable across replicates for both array formats. Many of the measurements have p-values ≤ 0.05, meaning that these measurements are significantly different from 1. These results indicate that most of these transcription factors are likely over-expressed at the dry seed stage, as compared to the reference. Where the two array formats disagree, for example, for transcription factors 13 and 17 in Figure 3, the result from Array Format 2 is likely more reliable as it contains 40 within-slide replicates of each oligo represented as opposed to only one within-slide spot of each cDNA in Array Format 1 that assayed a higher number (27,609) of unique cDNAs. Table 2 reports the p-values and standard errors for the 19 transcription factors of interest shown graphically in Figure 3 for both array formats. Based on the over-expression of most of these transcription factors in the dry seed, we conclude that the mRNAs and possibly the protein products of these transcription factors are produced late in seed development and then stored in the seed for use during the early stages of imbibition and germination.

**Table 2:** Expression data for selected transcription factors in both array formats.

			Array Format 1			Array Format 2		
**Figure 3^a^**	**Array Format 2 ID**	**Anno^b^**	**Ratio^c^**	**P-value^d^**	**SE^e^**	**Ratio^c^**	**P-value^d^**	**SE^e^**

1	SB0018	AP2	2.072	0.231983	0.409	52.887	<0.0001	6.521

2	SB0057	bHLH	2.001	0.011171	0.018	3.386	0.0002	0.562

3	SB0056	DNA-bdg	2.319	0.182539	0.389	1.089	0.5504	0.147

4	SB0044	fibrillarin	2.196	0.074646	0.141	4.129	<0.0001	0.160

5	SB0007	Hap	3.027	0.071495	0.229	1.274	0.0017	0.079

6	SB0091	Hap	2.000	0.217781	0.356	2.152	0.0011	0.276

7	SB0001	CBF-A	2.000	0.217781	0.356	2.518	0.0031	0.454

8	SB0020	PIF	3.658	0.076532	0.321	1.110	0.5013	0.161

9	SB0049	SCR	2.081	0.101934	0.175	1.433	0.0533	0.216

10	SB0085	SCL3	2.081	0.101934	0.175	2.242	0.0132	0.392

11	SB0054	SCR/Hat	2.702	0.020638	0.055	1.774	0.078	0.422

12	SB0086	SEUSS	2.091	0.2248	0.402	1.145	0.0294	0.064

13	SB0022	TIF	4.047	0.06914	0.332	0.972	0.8424	0.139

14	SB0066	TIF	4.047	0.06914	0.332	1.198	0.1634	0.137

15	SB0002	Tubby	2.256	0.149746	0.301	2.548	<0.0001	0.263

16	SB0047	AGP4	2.389	0.194411	0.438	3.314	<0.0001	0.317

17	SB0009	ZIM	2.008	0.015291	0.024	0.911	0.1933	0.067

18	SB0092	ZIM	2.008	0.015291	0.024	1.725	<0.0001	0.124

19	SB0068	zipper	2.860	0.009257	0.027	11.114	<0.0001	0.563

The average normalized ratio, p-value, and standard error in Array Formats 1 and 2 of 19 transcription factors is shown, at the stage of dry seed vs. 100-200 mg cotyledons. These values are displayed in graphical form in Figure 3, where the transcription factors are numbered according to the list in the first column. Also shown are the identification numbers of the gene in Array Format 2 and a brief annotation. In Array Format 2, different oligos may correspond to the 5' and 3' ends of the same gene; these genes' Array Format 1 values will thus appear twice.

a: Number given to gene in Figure 3.

b: Brief description of the gene's function, based on the longer annotation.

c: Average normalized ratio for that gene, in Array Format 1 or 2, at the dry seed stage (versus reference).

d: P-value for that gene at this stage. P-values less than 0.0001 are indicated with "<0.0001."

e: The standard error of the normalized ratios.

### Transcription factor mRNAs are expressed late in development

**Figure 4:**
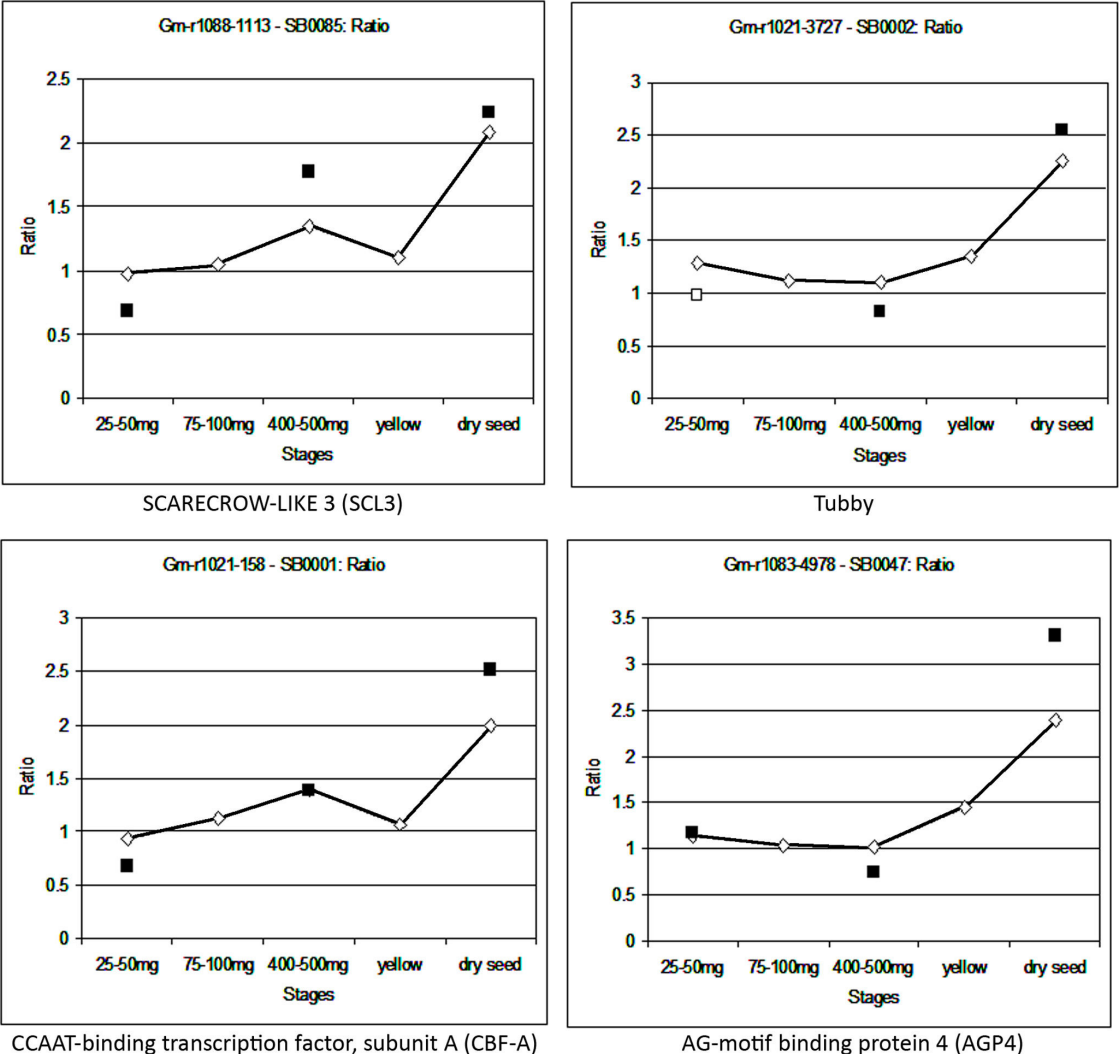
**Expression data from four transcription factors across five stages and two array formats**. X-axes show the five stages of development, each compared to the reference tissue. Y-axes show the ratio of the expression in the developmental stage compared to the reference. Diamonds (connected by lines) indicate the ratio according to Array Format 1. Squares indicate the ratio according to Array Format 2. Solid markers indicate measurement has p-value ≤ 0.05. All measurements shown have standard error ≤ 0.5. The name of the gene is shown below each graph. Note that scales differ slightly across graphs.

Figure 4 shows four of the nineteen transcription factors in individual graphs across all five stages of development, with the ratio data from both array formats. Although the ratios determined from Array Formats 1 and 2 are not usually exactly the same, both array formats show the gene following the same trend of expression over time. The reasons for the differences could be differences in the number of family members recognized by the Array Format 1 cDNA arrays versus the Array Format 2 oligo arrays since the cDNA arrays contain large portions of the coding regions while many of the 70-mer oligos may hybridize with fewer gene family members, generally 2-3 for the 19 transcription factors studied (see Methods).

SB0002 is annotated as a Tub family member in *Oryza sativa*. The Tub or tubby domain was characterized in mice as involved in controlling obesity [22] and is now found in a wide variety of eukaryotes, including humans, other animals, and plants [23]. There are a number of genes in the Tub family in various plant species, with fourteen *Tubby-like *(*TULP*) genes identified in rice [24] and eleven in *Arabidopsis *[25]. The specific functions of different *TULP *family members have yet to be determined in most cases; however, they frequently contain an F-box domain, suggesting they are ultimately involved in the ubiquitination of proteins selected for degradation in a wide variety of biological processes [24]. This is consistent in the current study with the large number of protein degradation-related genes that were found in sets containing genes that peaked at the dry seed stage. Cai et al. (2008) [26] found a *Tubby-like *gene in rice was involved in regulating a disease response gene. In addition to--or as part of--this role in transcriptional regulation, some *TULP *genes in both *Arabidopsis *and rice may be involved in signaling through abscisic acid and gibberellin pathways [23,25]. The over-expression of this gene in the dry seed stage compared to earlier green stages could be indicative of the protein degradation occurring as the seed desiccates and becomes quiescent.

SB0001 is annotated as a CCAAT-binding transcription factor, subunit A (*CBF-A*), in *Oryza sativa*. *CBF-A *is also known as Heme Activator Protein 3 (*HAP3*) and Nuclear Factor Y-B (*NF-YB*). Its protein constitutes one-third of the *HAP *complex which binds to the CCAAT-box element in the promoter of a gene; this element is very common in the promoters of genes from animals, fungi, and plants [27]. Animals and yeast have only one gene for each of the three subunits, but many genes for each subunit are found in plants. For example, there are ten genes for *HAP3 *in *Arabidopsis *[28] and eleven in both rice [27] and wheat [29]. *LEAFY COTYLEDON1 *(*LEC1*) is a well-studied *HAP3 *gene in *Arabidopsis *that has been shown to be involved in embryogenesis [30]. However, it is widely believed that the relatively large number of genes for the different *HAP *subunits in plants evolved to regulate transcription of genes in a variety of biological processes [31]. The function of just the *HAP3*-encoding genes has been linked to processes such as chloroplast formation in rice [32], improved yield in corn under drought stress [33], and flowering time in *Arabidopsis *[34]. Kwong et al. (2003) [35] and Yang et al. (2005) [31] divided *HAP3 *genes into two classes based on their similarity to *LEC1*, with the gene represented by SB0001 (EST accession # AI442376.1) falling into the "Non-*LEC1*-type" grouping, meaning it is likely to be involved in a process other than embryogenesis, for example protein degradation or desiccation tolerance.

SB0047 is annotated as AG-motif binding protein 4 (*AGP4*) in tobacco, which was first identified by Sugimoto et al. (2003) [36] during their investigation of *AGP1 *as a transcriptional regulator of a wound-inducible *Myb *transcription factor. They revealed the *AGP *family as GATA-type zinc finger proteins, transcription factors found in animals, fungi, and plants [37]. Members of this particular class of zinc finger proteins has been found to be involved in regulating a wide variety of genes in plants, including those responsive to light and circadian rhythms [37]. Other research into these GATA-type zinc finger proteins has shown them to affect nitrogen and sugar metabolism [38], cell elongation [39], and flower and shoot apical meristem development [40]. Given that Liu et al. (2005) [41] indicated that members of this family of transcription factors was involved in seed germination, it is possible the product of this gene is being accumulated in the seed during desiccation for later use during imbibition and germination.

SB0085 is annotated as *SCARECROW-LIKE 3 *(*SCL3*) in *Arabidopsis thaliana*. This gene was first identified by Pysh et al. (1999) [42] as part of a family of transcription factors known as *GRAS*, after *GIBBERELLIC ACID INSENSITIVE *(*GAI*), *REPRESSOR OF GA1 *(*RGA*), and *SCARECROW *(*SCR*). This family of genes has been identified in a wide variety of plants, including *Arabidopsis*, rice, maize, pea, oat, alfalfa, tomato, watermelon, and *Brassica napus *and is believed to be plant-specific [42-45]. *GRAS *genes in general have been found to be involved in light and gibberellic acid signaling as well as the formation of the axillary shoot and root meristems [43,44]. The *SCARECROW*-like genes have been primarily studied for their role in root development, including cell division, cell differentiation, and root tip regeneration [44]. Additionally they have been identified as targets of a root-knot nematode peptide that stimulates root growth and also as targets of miRNAs in *Arabidopsis *[45,46]. *SCARECROW *itself has been found to be expressed in multiple tissues during embryo development in *Arabidopsis *and maize, particularly in the region where the root meristem is formed [47,48]. Possibly, the product of this gene may accumulate in the seed during desiccation for use during germination, perhaps during early processes in root or shoot development.

Our analysis points to interesting transcription factors expressed late in development at a stage not previously surveyed in soybean. A comprehensive study of earlier stages of soybean seed development, including laser capture microdissection of various tissues from globular, heart, and cotyledon-stage soybeans, is discussed in Le et al. (2007) [49].

### Storage proteins are under-expressed late in development

**Figure 5:**
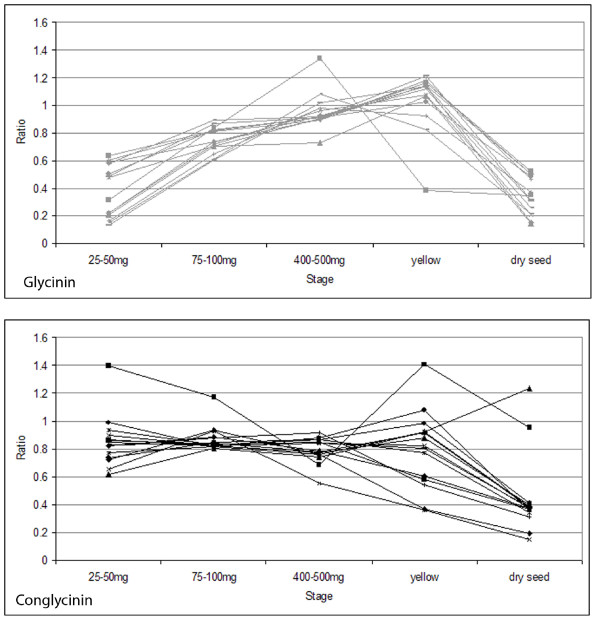
**Expression profiles of genes annotated as two storage proteins**. Data according to Array Format 1. Glycinin is shown at top, in grey; conglycinin is shown at bottom, in black. X-axes show the five stages of development, each compared to the reference tissue. Y-axes show the ratio of the expression in the developmental stage compared to the reference. All measurements shown have standard error ≤ 0.5. All fourteen genes annotated as glycinin, and fifteen genes annotated as conglycinin, from Array Format 1 are shown.

**Figure 6:**
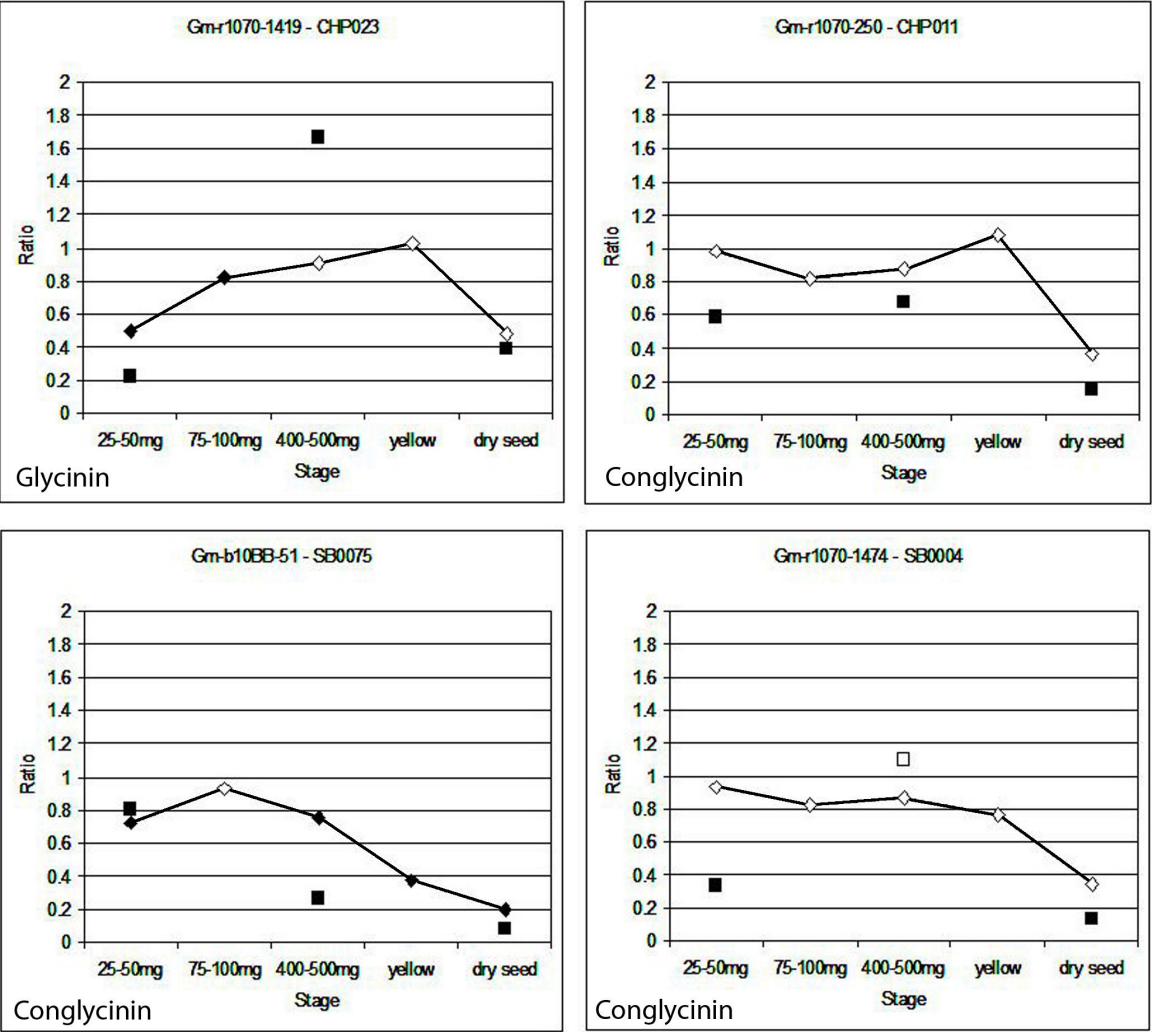
**Expression data from four storage protein genes across five stages and two array formats**. Genes annotated as glycinin (upper left) or conglycinin (other three). X-axes show the five stages of development, each compared to the reference tissue. Y-axes show the ratio of the expression in the developmental stage compared to the ratio. Diamonds (connected by lines) indicate the ratio according to Array Format 1. Squares indicate the ratio according to Array Format 2. Solid markers indicate measurement has p-value ≤ 0.05. All measurements shown have standard error ≤ 0.5.

Figure 5 shows the expression patterns of a number of genes annotated as two major storage proteins of soybean, conglycinin and glycinin, using Array Format 1. All data shown have standard error ≤ 0.5, indicating the repeatability of the results across replicates. They display expression profiles consistent with known information: they decrease in expression at the dry seed stage, compared to the reference. The conglycinin genes appear to be more consistently expressed throughout the first three (green) stages of development studied here, while the glycinin genes are under-expressed at the first stage (25-50 mg) and rise steadily in expression after that. Nielsen et al. (1989) [50] noted that glycinin mRNAs were first detectable in soybean embryos around the cotyledon stage of development (prior to the stages examined in this part of the project), peaked during mid-maturation, then became undetectable by the stage of seed maturation and desiccation. The peak in glycinin gene expression may coincide with the stage used as reference tissue in this experiment (100-200 mg fresh weight), which may explain why the glycinin genes shown in Figure 5 tend to have ratios less than 1. Meinke et al. (1981) [51] noted similar results with genes encoding various subunits of conglycinin, with mRNA accumulation beginning in seeds during the cotyledon stage but undetectable in mature, desiccating seeds. Figure 6 shows four of the genes annotated as glycinin or conglycinin from Figure 5, but in individual graphs with the ratio data from both array formats. The data from both array formats show each gene following the same general trend of expression over time, especially the decrease in expression level at the dry seed stage compared to the reference. This result for the storage proteins contrasts directly with the transcription factors that are over-expressed in late development as shown in Figures 3 and 4.

## Conclusions

Arrays spotted with 27,609 cDNAs from soybean were used to obtain data on the gene expression changes over five stages of soybean cotyledon development, as compared to a reference stage. These stages include those when the seed is accumulating water and nutrients; the stage of highest fresh weight; a yellow, desiccating stage; and a dry, hard seed stage. A variety of expression patterns were found among the significant genes over these stages, including many whose expression peaked (compared to the reference) during the desiccating and dry seed stages. Many of these expression patterns and ratios were supported by additional experiments involving a second, highly-repetitive microarray format.

Genes with annotations related to cell wall development, protein folding, and energy production were commonly found to have expression profiles peaking in expression (compared to the reference) at the youngest stage studied, as would be expected with green, rapidly developing seeds. At the stage of highest fresh weight, before the seed begins to desiccate, genes with annotations in the seed proteins category were commonly found to peak in expression. A number of genes annotated as auxin down-regulated were also found to peak in expression at this stage. Surprisingly, many genes were found to peak in expression at the desiccating and dry stages of development, with annotations related to protein degradation, transcription factors, and other processes. The products of these genes may be used immediately by the seed to prepare for quiescence or may be accumulated for later use during imbibition and germination.

## Methods

### Plant material

Immature soybean seeds (*Glycine max *cv. Williams) were harvested from greenhouse-grown plants, sorted by the fresh weight ranges as shown in Figure 1, dissected to separate the seed coat from the cotyledon, then lyophilized. Dry seeds were harvested at maturity and stored at room temperature. Total RNA was extracted from immature cotyledons and mature dry seeds using phenol:chloroform and a lithium chloride precipitation [14]. Soybean is highly inbred, but in order to minimize biological variation, RNA was extracted from approximately 10 to 30 seeds (depending on the stage) from multiple plants.

### Construction of microarrays

Details of construction and use of Array Format 1 have been reported [9,13]. Briefly, ESTs from libraries representing a variety of soybean tissues were contigged to identify unigenes, then clones representative of about 27,609 unigenes were re-racked to build three new libraries. The 3' ends of the unigenes were sequenced. Purified PCR products of the three libraries were single-spotted on amine slides (TeleChem International, Sunnyvale, CA) using a Cartesian PixSys 8200 arrayer (Cartesian, Irvine, CA). The set of 27,609 soybean cDNAs for Array Format 1 also includes 64 choice clones that were each printed 24 times (eight times from each of three libraries).

To construct Array Format 2, 192 70-mer oligos based on cDNAs from Array Format 1 were designed and synthesized (Illumina/Invitrogen, Inc., San Diego, CA). These oligos were designed where possible to represent the 3' end of the corresponding cDNA due to the higher sequence variability within this region. The oligos were designed to represent a cluster of EST sequences and were designed from a single EST representative, not from a consensus sequence. The 192 sequences are part of a larger set of 38,400 oligos that represent a soybean unigene collection [14]. However, since soybean is an ancient autotetraploid, many oligos will hit 2 to 3 members of highly related gene families or paralogous sequences. Each of the 192 oligos was then printed forty times on each amine slide (Corning GAPS II slides, Acton, MA) using a Genetix QArray2 robot (Hampshire, UK).

### Hybridization reactions and replicates

For both array formats, the RNA was hybridized to the microarray slides using a direct-label two-color dual hybridization procedure using Cy3-dUTP or Cy5-dUTP [13,52]. Approximately 80 μg total RNA was used with Array Format 1 and 40 μg with Array Format 2. The slides were scanned using a ScanArray Express (Perkin Elmer Life Sciences, Boston, MA) for Array Format 1 or a GenePix 4000B (Molecular Devices Corp., Sunnyvale, CA) for Array Format 2. The spots were found and their fluorescence intensity levels quantitated using ScanArray Express or GenePix Pro 6.0 software, respectively.

For Array Format 1, four slide replicates were made from each of the first three green stages (25-50 mg, 75-100 mg, 400-500 mg) including two dye swaps to mitigate any dye bias. The amount of material available from the later stage of the desiccating, yellow seed at 200-300 mg weight range was limiting at the time and the RNA yields are lower from the older seed, so for the final two stages only two slide replicates from each stage were made, also incorporating a dye swap. In all cases, the 100-200 mg fresh weight range served as the reference RNA in the two-color hybridization reactions. For Array Format 2, two slide hybridization replicates were made for each of the three stages (25-50 mg, 400-500 mg, and dry seed) and again compared to the 100-200 mg reference, including a dye swap. Independent biological samples were used for Array Format 2 compared to Array Format 1.

Because the average size of inserts on the Array Format 1 cDNA arrays is 1.3 kb [9], mRNAs of all family members with 85% similarity will likely be detected. Likewise, the 70-mer oligos of Array Format 2 also will hybridize to mRNAs with regions of sequence similarity of 85% over 20 nucleotides or more. Thus, they are not likely to distinguish all family members or paralogous genes since soybean is an ancient autotetraploid. For example, BLAST results of the 70-mer oligos representing the 19 transcription factors shown in Table 2 to the recently completed soybean genome sequence [53] showed that the majority hit only 2-3 genomic locations.

### Data analysis

The p-values were calculated by GeneSpring using a one-sample, two-tailed t-test with the hypothetical mean set to 1. Due to multiple functions listed in the annotations, or to different functions attributed to the 5' end vs. the 3' end, some clone IDs were placed in two or more categories. These clone IDs were *not *considered when calculating the percentage of cDNAs in each functional category for each cluster. The data from Array Format 2 were normalized by GeneSpring GX using a Lowess normalization procedure, with each spot counted individually. Data from both replicate slides were averaged together for each spot. The ratios of the forty spots representing the same gene were then averaged together using GraphPad [54], meaning each gene is represented by a total of eighty replicate measurements. GraphPad was also used to calculate p-values and standard error for Array Format 2 data.

For Array Format 1, the *k*-means algorithm in GeneSpring was applied to randomly separate the genes into the number of clusters defined by the user (in this case, eleven). The centroid of each cluster was calculated by averaging the coordinates attached to each gene. Each gene was then reassigned to the centroid to which it was closest and the coordinates of the centroids were recalculated. This operation was performed numerous times until the data converged, resulting in the clusters shown in Figure 2. Array Format 1 data have been deposited in the NCBI Gene Expression Omnibus (GEO) database as accession number GSE18620.

## Authors' contributions

SIJ designed the experiments; performed hybridizations, data analysis, and interpretation; and drafted the manuscript. DOG designed and printed the cDNA and oligo arrays. LOV designed approaches; led and coordinated the project; and edited the manuscript. All authors have read and approved the final manuscript.
